# Needs assessment and impact of COVID-19 on pharmacy professionals in 31 commonwealth countries

**DOI:** 10.1186/s40545-020-00275-7

**Published:** 2020-10-21

**Authors:** Diane Ashiru-Oredope, Amy Hai Yan Chan, Omotayo Olaoye, Victoria Rutter, Zaheer-Ud-Din Babar, The C. P. A. COVID-19 Action Team, Claire Anderson, Raymond Anderson, Manjula Halai, Ayodeji Matuluko, Winnie Nambatya, Chloe Tuck, Rao Vadlamudi, Hayley Wickens

**Affiliations:** 1 Commonwealth Pharmacists Association, London, UK; 2 School of Pharmacy, Faculty of Medical and Health Sciences, University of Auckland, Level 3, Building 505, 85 Pard Road, Grafton, Auckland, 1023 New Zealand; 3 Department of Pharmacy, University of Huddersfield, Queensgate, Huddersfield, HD1 3DH UK

**Keywords:** Pharmacy, Pharmacists, Commonwealth, COVID-19, Health, Heads of Government, Pandemic, CHOGM, CPA, Emergency preparedness, Coronavirus, CwPAMS

## Abstract

**Background:**

The declaration of COVID-19 a pandemic by the World Health Organization on 11 March 2020 marked the beginning of a global health crisis of an unprecedented nature and scale. The approach taken by countries across the world varied widely, however, the delivery of frontline healthcare was consistently recognised as being central to the pandemic response. This study aimed to identify and explore the issues currently facing pharmacy teams across Commonwealth countries during the COVID-19 pandemic. The study also evaluates pharmacy professionals’ understanding of key knowledge areas from the COVID-19 webinar hosted by the Commonwealth Pharmacists’ Association (CPA).

**Method:**

A quantitative survey-based approach was adopted, using a 32-item questionnaire developed from the literature on pharmacy and pandemic response. The survey was hosted on Survey Monkey and pilot tested. The final survey was disseminated by CPA member organisations. A 6-item online questionnaire was sent via email to all attendees of CPA's COVID-19 webinar. Descriptive statistics on frequency distributions and percentages were used to analyse the responses. Data were analysed using Microsoft^®^ Excel (2010).

**Results:**

There were 545 responses from pharmacy professionals across 31/54 Commonwealth countries in Africa, Asia, the Americas, Europe and the Pacific. Majority of the respondents reported being at least somewhat worried (90%) and more than 65% were very worried or extremely about the impact of COVID-19 on them personally and professionally. Nearly two-thirds of respondents stated finding it somewhat difficult or very difficult to work effectively during the pandemic. Challenges mostly faced by pharmacy professionals working remotely included; general anxiety about the impact of COVID-19 on their lives (12%), and difficulties in communicating with their co-workers (12%). Most pharmacy professionals had not previously been actively involved in a global health emergency (82%) nor obtained training on global/public health emergency preparedness (62%). Between 45 and 97% of the COVID-19 webinar attendees provided the correct answers to post-webinar questions, suggesting some improvement in knowledge.

**Conclusion:**

Our study confirms pharmacy professionals’ concerns about practice during a pandemic and provides preliminary data on the challenges and learning needs of the profession. The CPA has since acted on these findings, providing ongoing opportunities to develop and refine resources for the profession as the pandemic evolves. Pharmacy professionals have also demonstrated improved knowledge on the management of COVID-19 and resources available for professionals.

## Background

COVID-19 was declared a pandemic by the World Health Organization (WHO) on 11 March 2020, marking the beginning of a global health crisis of an unprecedented nature and scale [1]. The response to the pandemic by countries across the world varied widely, with differences in the type of strategy and timing of steps employed. Regardless of the approach taken, the delivery of frontline healthcare was consistently recognised as being central to the pandemic response [2]. There were significant changes in healthcare delivery as a result of lockdown restrictions, such as travel and social distancing restrictions, and reductions in workforce capacity and/or working hours [3]. With the increased pressure on the health system, pharmacists were called upon as key members of the healthcare team to support and alleviate the burden on overcrowded emergency departments and free up medical staff to treat more unwell patients [4]. In many countries, at a national level, pharmacies were defined as essential services, and were one of the few services that remained opened and accessible to the public when countries were placed in lockdown with recommendations in some countries for pharmacies to stay open 7 days a week and during usual holidays to manage the COVID-19 pandemic [5, 6].

Pharmacy teams are critical members of the healthcare workforce and are considered essential frontline healthcare workers. The pharmacy workforce provides a wide range of healthcare services in community pharmacies; healthcare hubs designed to meet health needs in communities [7, 8]. As healthcare professionals, they were strategically positioned help manage the COVID-19 pandemic and strengthen a country’s pandemic response and readiness through their roles in the community. Pharmacists are also easy to access healthcare professionals and community pharmacies are more widely distributed in some countries than supermarkets, banks and medical centres [4]. Pharmacies are also familiar places to the community, and most community pharmacists and pharmacy team members have established relationships with the public and with primary care providers and are therefore ideally positioned to support other healthcare providers in the pandemic response. The dual role of pharmacists as healthcare providers and retailers [9] also brings opportunities for flexibility in healthcare service delivery models during health crises. Pharmacists can dispense medicines and ration supplies, administer vaccinations, but also supply consumables and health-related products [8, 9].

A strong evidence base exists showing that pharmacists are in a prime position to support a pandemic response [10], yet their skill sets are often not recognised and underutilised [4]. Anecdotal evidence suggests that the support available in each country for pharmacy teams have varied widely with pharmacists receiving differing levels of guidance on how to manage COVID-19 and how to adequately prepare their pharmacy teams as part of the pandemic response, particularly as “gatekeepers with no safety” [11]. Pharmacists in the UK and Pakistan collaborated to compile guidelines for pharmacy teams in English and Urdu in an attempt to assist the response [12]. In some countries such as New Zealand and Northern Ireland, the government has acknowledged pharmacists’ contribution during the pandemic by providing extra remuneration [12, 13]. The New Zealand Ministry of Health also produced useful resources for pharmacists including posters on infection prevention, information sheets to educate the general public about COVID-19, and educative social media images [14].

The Commonwealth Pharmacists Association (CPA) has been uniting and supporting the pharmacy workforce throughout the Commonwealth during these challenging times. The Commonwealth is a voluntary association consisting of 54 independent and equal countries spanning every continent. It is home to 2.4 billion people; approximately a third of the world’s population and includes both advanced economies and a high proportion of lower-resourced countries, including many island nations. Member governments share goals such as development, democracy and peace [15]. The CPA is a registered charity whose vision is to “empower pharmacists to improve health and wellbeing throughout the Commonwealth”. Its mission is focused on facilitating better access to medicines and improving the quality of, and safer and more effective use of medicines in the Commonwealth, particularly in lower-middle income countries (LMICs). The CPA achieves this through building strong collaborative networks; partnering with member organisations to improve the quality of pharmacy practice; and creating platforms for the dissemination of knowledge about pharmaceutical sciences and professional practice. The Commonwealth and the CPA are therefore well-placed to play a pivotal role in the pandemic response [16]. As a globally focused civil society organisation, it is critical that the CPA responds to actual rather than perceived needs. However, there is limited data currently available to describe the issues affecting the ability of the pharmacy profession to optimally respond to the pandemic, and the gaps that exist in information provision.

In this context, this study was planned to identify and explore the issues that pharmacy teams across Commonwealth countries faced during the COVID-19 pandemic, specifically:

- To evaluate how concerned pharmacy staff are about COVID-19 and their ability to work effectively during the COVID-19 pandemic;
- To explore what work has been done by pharmacy professionals and/or professional bodies as part of the current pandemic response;
- To analyse what support would be helpful to receive to better equip the profession to respond to the pandemic.
- To measure the impact of COVID-19 webinars on participant’s knowledge of COVID-19 as well as resources available for pharmacy professionals across the commonwealth.

## Methods

### Survey development

A quantitative survey-based approach was adopted using a 32-item questionnaire developed from the literature on pharmacy and pandemic response. This questionnaire was not validated for reliability and validity as the questionnaire needed to be responsive to the evolving pandemic in a timely manner, however questions were informed by current literature on pharmaceutical and pandemic responses. All questions were presented to respondents in English language. Most questions were in Likert-scale response format, with categorical responses ranging from ‘extremely’ to ‘not at all’, depending on the focus of the question. In some questions, respondents were invited to select more than one option from a list of possible responses, for example, to identify the challenges that pharmacy professionals were facing during the pandemic, respondents were shown a list of challenges from which they could select their top three challenges (see Additional file 2 for the full questionnaire).

Demographic data were also collected from respondents, including information on age, gender, country, number of years of practice in their current profession, and predominant work setting. The survey was reviewed and refined by discussing with a working group comprising members from the CPA COVID-19 action team with expertise covering pharmacy, infection management, health psychology, global health, and healthcare communications.

The survey was then hosted on Survey Monkey (surveymonkey.com, USA), a web-based survey platform, then pilot tested with eight individuals across UK, Uganda and New Zealand. Following this initial testing, the final survey was disseminated by CPA member organisations (national pharmacy organisations) via the CPA members list and associated networks. The survey was also promoted via CPA social media platforms, including LinkedIn, Twitter, and Facebook, and the CPA newsletter and website (https://thecommonwealth.org/about-us).

Further details on the methodology including the process for development and pre-testing are provided in Additional file 1 which completes the CHERRIES checklist for web-based studies [17]. The survey questionnaire is also provided as Additional file 2.

### CPA COVID-19 webinar

The CPA hosted its first webinar on COVID-19 on 7 May 2020. The aim of the webinar was to find out how pharmacy teams across the Commonwealth had been coping with the pandemic as well as provide information on COVID-19 resources available for pharmacy professionals. As part of the evaluation of the webinar, a 6-item online questionnaire was designed and sent to webinar attendees. The questions comprised of three multiple-choice questions and three close-ended statements/questions to assess key knowledge areas (e.g. treatments for COVID-19) that were discussed in the webinar. A question in a Likert-scale format was designed to assess attendee’s satisfaction with the webinar. The survey was then sent to webinar attendees via email.

### Respondent eligibility

#### CPA COVID-19 survey

Pharmacy professionals (pharmacists, pharmacy technicians and dispensers) across the 54 Commonwealth countries were the intended audience for completion of the survey. Participation was voluntary, with the questionnaire being open for responses over a 4-week period (25 March until 26 April 2020).

#### CPA COVID-19 webinar

All webinar attendees across the 54 Commonwealth countries were eligible to complete the survey. Participation was voluntary, with the questionnaire being open for responses over a 7-day period (14 May until 20 May 2020).

### Data management

Data were collected anonymously, although survey respondents could voluntarily provide their name and email address should they wish to be contacted following the survey with information related to the survey or other COVID-19-related information. The data were held securely and in-line with the General Data Protection Regulation 2016/679 [18].

Approval to carry out the study was obtained by the CPA board of trustees. Ethical approval was not required because this is a service evaluation. All respondents participated strictly in their professional capacity, and gave informed consent to participate in the survey.

### Data analysis

Descriptive statistics on the frequency distributions and percentages were used to analyse the responses. Data were analysed using Microsoft^®^ Excel (2010).

## Results

### Demographics of respondents

Overall, there were 545 responses from pharmacy professionals (486 pharmacists and 59 pharmacy technicians) across 37 countries; 31 of which are Commonwealth countries (Table 1). Additionally, there were responses from 111 pharmacy students from 7 countries. India (76), Tanzania (27), Bangladesh (2), Uganda (2), Zambia (2), Pakistan (1) and the UK (1). Over half of the respondents were in the age range of 25–44 years, with the majority having less than 10 years of experience. Most respondents (71%) also worked in either a hospital or in a community setting.

**Table 1:** Country distribution of responses from pharmacy professionals (pharmacists and pharmacy technicians), (*n* = 545)

Country	Pharmacist and pharmacy technician	Percentage (%)
African Region: Rwanda, Tanzania, Ghana, Zambia, Uganda, Nigeria, Kenya, Cameroon, Malawi, South Africa, Mauritius, Swaziland, Zimbabwe	298	54.8
Western Pacific Region: Australia, Malaysia, Singapore, Fiji, New Zealand, Samoa	110	20
Region of the Americas: Canada, Dominica, Saint Vincent and the Grenadines, Trinidad and Tobago, United States of America^a^, Grenada, Saint Lucia, Guyana	49	9
South-East Asia Region: India, Nepal^a^, Sri-Lanka	43	8
European Region: Republic of Ireland^a^, United Kingdom, Malta	27	5
Eastern Mediterranean Region: Afghanistan^a^, Pakistan, United Arab Emirates^a^, Jordan^a^	17	3
Unknown	1	0.2

	Number of respondents	(%)
**Age (years)**		
18 to 24	43	7.9
25 to 34	188	34.5
35 to 44	132	24.2
45 to 54	50	9.2
55 to 64	24	4.4
> 65	8	1.8
No response	98	18.0
**Gender**		
Male	272	50.0
Female	254	46.1
No response	18	3.3
Prefer not to say	1	0.2
**Years in profession**		
< 1	37	6.8
1–3	96	17.6
4–10	143	26.2
11–15	75	13.8
> 15	108	19.8
No response	86	15.8
**Professional setting**		
Community	216	39.6
Hospital	172	31.6
Academia (University (as an academic) or research institute)	77	14.1
Government (local, regional or nationally)	23	4.2
Industry	18	3.3
Public Health Institute	17	3,1
Professional body	10	1.8
Others (Post graduate students, military, did not specify)	8	1.5
Non-governmental organisation	3	0.6
No response	1	0.2

^a^Indicates non-Commonwealth countries

## Survey findings

### Level of concern about COVID-19 and ability to work effectively during the pandemic

**Fig. 1:**
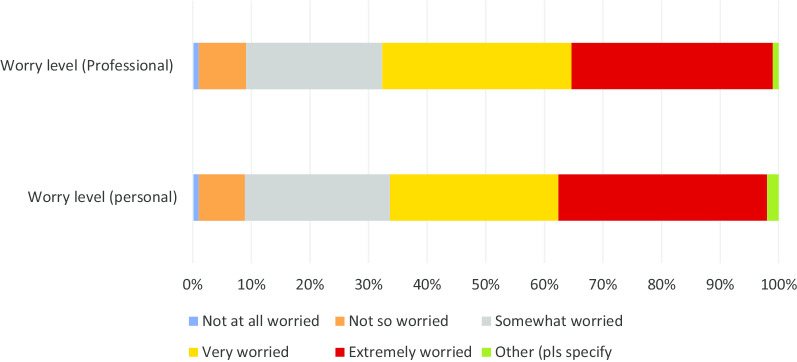
How worried respondents were on the impact of COVID-19 on them personally and the pharmacy profession in their country (*n* = 545)

Figure 1 illustrates the level of worry from respondents about the impact of COVID-19 on them personally and the pharmacy profession. More than 90% of people reported at least being somewhat worried, with nearly two-thirds reporting being ‘very worried’ or ‘extremely worried’. Extreme worry was observed to be higher on a personal level than on a professional level.

### Impact on effective working and need for remote working

**Fig. 2:**
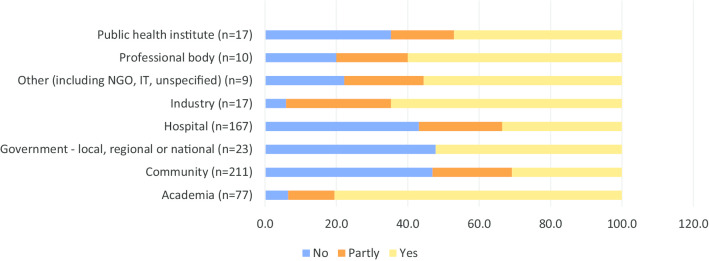
Percentage of respondents reporting needing to work remotely, according to work setting (*n* = 531)

Nearly two-thirds of respondents stated finding it somewhat difficult or very difficult to work effectively during the COVID-19 pandemic. Figure 2 illustrates the percentage breakdown of respondents who have needed to work remotely, by work setting. This shows that respondents working in academia, professional bodies and industry are more likely to work remotely compared to those in government and patient-facing roles such as community and hospital.

### Key challenges with remote working

The most common challenges that pharmacy professionals selected with remote working (Table 2) were: general anxiety about the impact of coronavirus on their life, and difficulties with communication with their co-workers (each selected by 12% of respondents), issues with internet connectivity, social isolation, keeping a regular schedule, no access to tools or information needed to work at home, and issues with physical work space (each selected by 11% respondents). Challenges that were least faced were childcare (4.5%), getting enough food (1.8%) and being sick, or helping the sick (1.4%).

**Table 2:** Top three challenges facing pharmacy professionals with remote working (*n* = 545)

Challenges currently faced by pharmacy professionals with remote working	Number	Percentage (%)
General anxiety about the impact of coronavirus on my life	173	12.2
Communication with co-workers is harder	170	12.0
Internet connectivity	158	11.2
Social isolation	156	11.0
Keeping a regular schedule	155	11.0
I don’t have access to the tools or information I need to do my job at home	153	10.8
My physical workspace	149	10.5
Too many distractions at home	141	10.0
Childcare	64	4.5
Other (please specify)	48	3.4
Getting enough food	26	1.8
I’m sick or helping others who are sick	20	1.4
	1413	

### Impact of social distancing on work

There was a mixed response on the impact of social distancing on the pharmacy profession with 28% of respondents stating it had significantly increased workload and visits to the pharmacy, yet a similar proportion (28.9) reported a reduced workload and visits to the pharmacy (Table 3).

**Table 3:** Impact of social distancing on workload of pharmacy profession (*n* = 440)

Impact of social distancing on pharmacy	Number	%
A slight increase in workload and visits to the pharmacy	76	17.3
Don't know	28	6.4
Not much impact on workload or visits to the pharmacy	48	10.9
Other (please specify)	38	8.6
Reduced workload and visits to the pharmacy	127	28.9
Significantly increased workload and visits to the pharmacy	123	28.0

### Work done by pharmacy professional and/or professional bodies in response to the pandemic

Majority of the respondents (*n* = 479) were aware of pharmacy organisation or a pharmacist who was involved/consulted in COVID-19 response or preparation directly (40%) or indirectly (30%). In contrast, 16% stated that they were not aware of any pharmacists or pharmacy organisation being involved/consulted in COVID-19 response; 13% did not know and 1% selected other for contributions, e.g. developing new workflow for medication extension, home delivery, managing adequacy of drug supplies due to global supply chain disruption and supporting/implementing tele-counselling.

More than a third of respondents (40%) stated that one or more COVID-19 responses had been spearheaded or proposed by pharmacy organisations in their country, a third were unsure (31%), 23% stated no responses had been spearheaded by pharmacy organisations and 6% selected sort of.

**Fig. 3:**
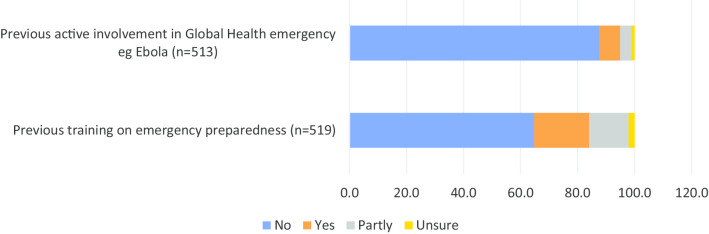
Percentage of respondents with previous involvement in a global health emergency (*n* = 522) or previous training on emergency preparedness (*n* = 518)

Most pharmacy professionals had not previously been actively involved in a global health emergency (82%) nor had training on emergency preparedness global/public health emergency preparedness (62%) (Fig. 3).

### Support to better equip the profession to respond to the pandemic

When asked what kind of support respondents would find helpful from the CPA, most selected from the suggested list of options webinars (28%), and access to community of support to share questions and concerns (26%) (Table 4). Signposting to information was only selected by 17% of respondents and 6% of respondents provided other suggestions which included, e.g. access to protective personal equipment, training, guidance on tele-consults by pharmacists for therapy management, support in conducting research and literature review.

**Table 4:** Support needed to better equip the pharmacy profession to respond to the COVID-19 pandemic (*n* = 545)

Support required	Number	%
Webinars on COVID-19	150	27.5
Access to a community of support to share questions and concerns	140	25.7
Signposting to information	90	16.5
Other suggestions	32	5.9

### COVID-19 webinar

The first webinar on COVID-19 was organised by the CPA and held on 7 May 2020. It was an opportunity to discuss resources already available to support the COVID-19 response throughout the Commonwealth. Easy access to these resources collated from the WHO, International Pharmaceutical Federation (FIP), Africa Centres for Disease Control (Africa CDC), Ministries of Health, and national pharmacy associations was provided through the Commonwealth Partnerships for Antimicrobial Stewardship Smartphone app (CwPAMS App). Future webinars are planned to provide a discussion forum and an opportunity to share learning based on experiences. This will be particularly important when testing, vaccination and treatment options are rolled out.

The webinar had 620 registrations from 38 countries. Top five registrations were from Nigeria (20%), Kenya (17%), Malaysia (11%), India (8%) and Pakistan (7%). A post-webinar feedback questionnaire was completed by 264 individuals; 71% were pharmacists and 19% pharmacy students. 75% watched the live session while 23% watched the recording. Majority of the respondents found the webinar very useful (64%) and useful (29%) (*n* = 259).

The feedback also included six knowledge quizzes (Table 5). One of which was on organisations that have developed international recommendations/guidelines on COVID-19. Responses revealed that 97%, 53% and 45% correctly identified that WHO, FIP and Africa CDC have developed international recommendations/guidelines on COVID-19. 24% of respondents correctly answered the question on relevant COVID-19 resources that have been developed or in development as part of the CwPAMS with ‘hand rub formulation training video’ and ‘app’ selected by 78% and 73% of respondents, respectively. In addition, some respondents incorrectly selected ‘COVID-19 Treatment guidelines’ (59%), ‘COVID-19 treatment’ (0.4%) and ‘hand rub formulation’ (0.4%). A total of 49% respondents correctly answered the question on the spread of COVID-19 identifying that COVID-19 is spread by droplets (96%) and surfaces (82%) while 34% incorrectly selected that the coronavirus is airborne.

**Table 5:** Percentage of respondents who answered each key knowledge question correctly (*n* = 264)

Key knowledge question (*n*)	Correct answer	Correct (%)	Incorrect (%)
1. Which organisations have developed international recommendations/guidelines on COVID-19?	WHO, FIP, Africa CDC	39	61
2. There are currently one or more treatments for COVID-19 that have been fully tested for safety and efficacy (False)	False	86	14
3. Chloroquine and hydroxychlorine can be used to treat COVID-19 outside of clinical trials	False	59	41
4. The CwPAMS app, currently piloted in Ghana, Uganda, Tanzania and Zambia has other international resources for AMR and COVID-19? (*n* = 18 356)	True	87	13
5. What COVID-19 relevant resources have been developed or in development as part of CwPAMS?	Hand rub formulation training video, App	24	76
6. Spread of Coronavirus is via?	Droplet, surface	49	51

## Discussion

This is one of the first evaluations of the impact of the COVID-19 pandemic on the global pharmacy profession and its ability to work effectively, and to identify what resources are needed to better support the profession. The study found high levels of worry amongst almost all respondents both on a professional and personal level with extreme worry observed to be higher on a personal level than on a professional level.

This correlates with a very recent study on the psychological impact of the COVID-19 pandemic on health care workers in a MERS-CoV endemic country which revealed that based on the 1–5 worry rating scale, healthcare staff were more anxious about personal implications of COVID-19 such as the transmission of the disease to a family member rather than acquiring the infection themselves [19].

Most respondents reported facing some difficulty with working effectively, with nearly half having to adjust to remote working, particularly those working in non-patient-facing roles such as in industry or academia. Major difficulties stated by respondents such as anxiety, communication and physical workspace were common to pharmacy professionals worldwide. However, from previous studies, specific challenges such as poor internet connectivity which could significantly affect access to tools or information needed to work at home are frequently associated with LMICs which constitute a significant part of the Commonwealth [18]. The impact on workload from social distancing was mixed with 28% of respondents stating it had significantly increased workload and visits to the pharmacy, yet a similar proportion reported a reduced workload and visits to the pharmacy. This can be explained by uncertainties associated with the pandemic. At the early stages of the pandemic, there was a major upsurge in workload initially when there was much uncertainty among populations regarding access to their medicines followed by a reduction when supplies were secured. This was however reassuring as most respondents were able to identify some level of involvement of pharmacy as part of a COVID-19 response. These findings are in line with the literature available on the role of pharmacy in managing health crises, which highlight the key benefits of partnerships between the pharmacy sector and government bodies in ensuring an optimal pandemic response [20–25]. A recent publication on Global Contributions of Pharmacists across eight countries namely; the United States of America, United Kingdom, Canada, Saudi Arabia, Qatar, South Africa, Lebanon and Nigeria during COVID-19 pandemic also highlights essential services offered by pharmacists within and outside of core their roles during the pandemic [26].

In terms of the needs assessment, most pharmacy professionals had not previously been actively involved in a global health emergency and did not have previous training on emergency preparedness. Respondents identified a need to up skill to better respond to the pandemic; they felt this would be best achieved through webinars on COVID-19, access to a community of support to share questions and concerns and being signposted to information among other suggestions. This resonates with a recent survey in India where pharmacy professionals expressed willingness to be trained and better equipped for COVID-19 and other health emergencies which could occur in the near future [20]. Based on these findings, the CPA rapidly developed a number of activities to support pharmacy professionals across the Commonwealth. These included:

### COVID-19 webinars

The webinar on 7 May 2020 was an opportunity to discuss resources already available to support the COVID-19 response throughout the Commonwealth [27]. Responses to key knowledge questions on COVID-19 revealed that the webinar significantly improved attendee’s knowledge on COVID-19 resources available for pharmacy teams, latest updates in the treatment of COVID-19 and the contents of the CwPAMS App. However, a higher percentage of respondents provided correct answers to close-ended questions than multiple-choice questions. This can be clearly explained by the multiple-choice question format as respondents were required to select all right options to be considered correct as opposed to close-ended questions which required the selection of only one option. Notably, there were varying responses to the question on the spread of COVID-19 as research is ongoing in this area. In a recent briefing from WHO, it was stated that the possibility of airborne transmission of the coronavirus in public settings, especially closed, poorly ventilated settings cannot be ruled out and emphasised WHO’s commitment in the provision of credible and accurate information concerning COVID-19 [28]. The CPA has planned future webinars to provide a discussion forum and an opportunity to share learning based on experiences. This will be particularly important when testing, vaccination and treatment options are rolled out.

Future webinars planned include shared learning panel webinars with pharmacists from countries across the Commonwealth discussing their response and challenges to tackling COVID-19.

### Implementation of resources/toolkits and advocacy because of the findings

COVID-19 community of support: a dedicated email address and a Telegram group was established to provide a discussion and support community to members. As part of the ‘CPA 2020 50th Birthday Challenge’ a virtual tour of the Commonwealth was launched (which commenced before COVID-19 was declared a pandemic). The CPA have used the opportunity to check in with member organisations to understand how they are coping and how the CPA could be of further support at this challenging time; this was also advertised on Twitter using the hashtag #CPA2020C.

COVID-19 resources toolkit: A webpage was added to the CPA website to signpost COVID-19 resources that were directly relevant to the pharmacy workforce—including international guidance, publications, tools and resources produced by national pharmacy member organisations. The CPA also produced a downloadable toolkit based on the infection prevention control (IPC) activities that were developed as part of the CwPAMS programme [29].

Easy to access COVID-19 resources via CwPAMS App: Another development which is linked to the CwPAMS programme has been the introduction of a COVID-19 section on the CwPAMS App that was developed to bring together resources to support IPC and antimicrobial stewardship at the point of care, independent of internet connection in four LMICs.

Advocacy: the CPA’s advocacy role has continued through the COVID-19 pandemic. For example, via the recent virtual Commonwealth Civil Society Forum, which traditionally takes place on the eve of the Commonwealth Health Ministers Meeting (CHMM) in Geneva each year. This year, the theme was how digital health could help support the response to the COVID-19 pandemic. The CwPAMS App was showcased and the CPA presented how the pharmacy workforce across the commonwealth is well placed to use digital technologies as part of the response to the COVID-19 pandemic, particularly supporting the access to quality medicines and prescribing information. These points were reflected in the statement and recommendations that were put forward to health ministers.

CPA has also contributed to the advocacy papers ahead of the (postponed) biennial Commonwealth Heads of Government Meeting (CHOGM) that was set to take place in Kigali, Rwanda, on 22–27 June 2020. These efforts seek to raise the profile of the profession alongside issues that need to be brought to the attention of policy-makers relating to medicines and progressing towards Universal Health Coverage (UHC). The theme for CHOGM 2020 is ‘Delivering a Common Future: Connecting, Innovating, Transforming’. The COVID-19 pandemic has brought new, unprecedented health challenges such as questions on how to ensure ongoing, equitable access to pharmaceutical care, and protect the safety of our current health workforce. This led to the CPA authoring a commentary that will be disseminated to policy-makers, entitled ‘The role of the Commonwealth in achieving UHC through pharmaceutical care amidst the COVID-19 pandemic’ [16].

A training video on local production of hand sanitisers based on the WHO formulation: As part of our advocacy efforts, the CPA secured funding from the Commonwealth Secretariat to produce a training video on local production of hand sanitisers using the WHO formulation. This was produced to help pharmacists in LMICs to produce these alcohol-based hand sanitisers at a low cost. The video, which provides a step-by-step guide on the preparation, labelling and storage of the alcohol-based formula, has been developed under the CPA’s CwPAMS programme.

## Strengths and limitations

This survey provides a high-level overview of the issues facing the pharmacy profession in the Commonwealth and the potential to provide guidance to better support the response of the profession. The survey was able to obtain responses from a large number of countries and capture the views of respondents in different parts of the Commonwealth. There was a high level of response from LMICs. The results however do not provide in-depth detail of the issues potentially facing the profession; further research to explore the barriers and facilitators is warranted. Additionally, the questions used to develop the survey were not validated as there was limited literature at the time of survey launch to inform questionnaire development. Furthermore, there was the possibility of recall and self-report bias from respondents as some questions addressed past events. We also do not know how participants in the CPA webinar and training applied their learning and what the impact was on practice. Further evaluation in future follow-up surveys is needed to explore this. Nevertheless, the survey provides useful initial data amidst a global pandemic of the pharmacy sector response and can be used to inform ongoing work and studies.

## Conclusion

The pharmacy profession played an essential role in the COVID-19 pandemic response, for example in ensuring ongoing medicines supply and medicines access, supporting public health measures, and assisting in case identification and management. It is crucial that global health organisations, such as the CPA, are able to support pharmacists during the pandemic by providing the needed guidance and advice. Our study confirms the high level of worry amongst the profession and provides preliminary data on the issues and learning needs of the profession. The CPA has since acted on these findings, with ongoing opportunities to continue to develop and refine resources for the profession as this unprecedented global crisis continues to evolve. Pharmacy professionals across the Commonwealth demonstrated improved knowledge on the management of COVID-19 and resources available for professionals following education training.

## Supplementary information

**Additional file 1.** Completed CHERRIES checklist for web-based studies for the survey.

**Additional file 2.** CPA impact of COVID-19 and Pharmacy Needs Assessment Survey Tool.

## Data Availability

The datasets during and/or analysed during the current study are available from the corresponding author on reasonable request.

## Abbreviations

CPA: Commonwealth Pharmacists’ Association
CHMM: Commonwealth Health Ministers’ Meeting
COVID-19: Corona virus disease 2019
CwPAMS: Commonwealth Partnerships for Antimicrobial Stewardship
CHOGM: Commonwealth Heads of Government Meeting
LMICS: Low- and middle-income countries
UHC: Universal Health Coverage
WHO: World Health Organization
