# Supplementary material for: Needs assessment and impact of COVID-19 on pharmacy professionals in 31 commonwealth countries
Source: J Pharm Policy Pract. 2020 Oct 21;13:72. doi: 10.1186/s40545-020-00275-7 (PMC7576098; doi:10.1186/s40545-020-00275-7)
Supplement: Supplementary file 1 — Additional file 1. Completed CHERRIES checklist for web-based studies for the survey. [file 40545_2020_275_MOESM1_ESM.docx]

# Additional file 1: CHERRIES checklist for web-based studies^1^

| **Item category** | **Checklist item** | **Description** |
| --- | --- | --- |
| Design |  | The target population was a convenience sample of pharmacy professionals across any of the 54 Commonwealth Countries. |
| Ethics | Ethics approval | As this was a needs assessment to consider what support pharmacy professionals needed as part of the COVID response, ethical approval was not required. The CPA trustees granted permission for the study to take place |
|  | Informed consent | All respondents participated strictly in their professional capacity, and their participation in the survey was in all cases based on informed consent. They were informed of the purpose of the study and that participation was voluntary and that their responses were anonymous. On the front page which included the introduction they were also asked to confirm they wished to participate in the survey.  Consent was indicated when respondents clicked yes to the consent question at the bottom of the introductory page and moved to the next page.  An estimate time (approximately 10 minutes) to complete the survey (based on the short pilot phase) was also provided on the survey introduction. Responses to questions were stored as well as completion data, start and end time.  Also, on the introductory page participants were provided with the study lead’s details |
|  | Data protection | Survey Monkey was used for the survey.  Some participants provided their email address if they wished to be contacted in the future in relation to the survey. This was not linked to survey results. The data collected through the survey tool is not accessible without account permissions to view the responses. The fully de-identified dataset was used for analysis and is kept on password protected computers. |
| Development and pre-testing |  | In March 2020, the study lead designed a short survey and invited key members of the CPA team and trustees to pilot and comment on the draft survey tool.  The initial phase of developing the survey tool involved reviewing the literature using specified search terms. Following the initial phases of survey tool development, the online survey tool was piloted by 8 pharmacy professionals from 3 countries across Europe, Pacific and Africa for additional comments on the content, interpretation of questions and time-scale for completion. Following the consensus process and pilot, the final survey including 32 questions was finalised and deployed. |
| Recruitment process | Open vs closed survey | This was an open survey. |
|  | Contact mode | When the survey tool was finalised, link to the survey was included in the organisations newsletter comprising of national pharmacy associations and individual members from across the commonwealth. The survey link was also added to the organisation website and promoted via social media (Twitter, Facebook and LinkedIn).  (Appendix 2 and 3) |
|  | Advertising the  survey | The link to the survey was advertised through the organisation webpage, communications cascaded via the councillors, and Twitter and email was used as the primary social media tool by various organisations to promote the survey. Individual councils were able to use their relevant online mailing lists which consisted of any pharmacy professionals |

| **Item category** | **Checklist item** | **Description** |
| --- | --- | --- |
| Survey administration | Web/email | This was a web-based survey. Responses were collected through the online survey platform. Responses were multiple choice, numeric, and open text. |
|  | Context | Signpost to the survey URL was available on the organisation website. The main audience for these webpages pharmacists, in particular country representatives or national leads. |
|  | Mandatory/voluntary | Voluntary. |
|  | Incentives | There were no incentives offered to individuals. |
|  | Time/date | The data was collected from *25 March to 26 April 2020 (following a two extension from initial*  *deadline advertised).* |
|  | Item randomisation | The items were not randomised or alternated – the same order of questions was received by all participants. |
|  | Adaptive questioning | Some questions and pages were conditionally displayed e.g. for those who did not work remotely |
|  | Number of items | There were 32 items displayed across 6 pages. Due to the adaptive nature of the questionnaire, not all respondents were presented with all items e.g. the questions/page specific to prescribers. |
|  | Number of screens | The full survey was distributed over 6 pages including the introduction page |
|  | Completeness check | Several questions were set as requiring a response especially the adaptive questions, however these also included a Don’t know/unsure option. Non-response options were provided. |

| **Item category** | **Checklist item** | **Description** |
| --- | --- | --- |
|  | Review step | Respondents were able to review and change their answers using a ‘back’ button before submitting their form |
| Response rates | Unique site visitor | IP addresses and cookies were not recorded to ensure confidentiality. Also it was expected that users were likely to respond from their workplace, where there were likely to be using shared devices. |
|  | Participation rate | In total, 545 responses from pharmacy professionals (486 pharmacists and 59 pharmacy technicians) across 38 countries. Additionally, there responses from 111 pharmacy students from 7 countries. India (76), Tanzania (27), Bangladesh, Uganda, Zambia (2) each, Pakistan and United Kingdom (1) each. |
|  | Completion rate | Of the 1270 pharmacy professionals who provided a response to the question on the introductory page and accessed the main part of section of the survey, 656 respondents from the 38 countries responded to the first non-demographic question and were subsequently included for analysis (52%). |
| Preventing multiple entries from same individual | Cookies used | No |
|  | IP check | No |
|  | Log file analysis | Not used |
|  | Registration | Entry to the survey was via a unique login provided to each invitee to the survey. |
| Analysis | Handling of  incomplete questionnaires | Only responses from respondents that completed the first question on page 2. |
|  | Questionnaires with atypical timestamp | There was no timeframe set or used as a cut-off point |
|  | Statistical correction | No statistical correction was made during analysis |

Eysenbach G. Improving the quality of web surveys: The Checklist for Reporting Results of Internet E-Surveys (CHERRIES). J Med Internet Res. 2004;6(3):1–6.

Appendix 1: Communications template and translation URLs

|  |  |
| --- | --- |

Appendix 2: Sample advert on a professional body’s webpage and newsletter to members

Appendix 3: Sample social media messages promoting the survey URL

| 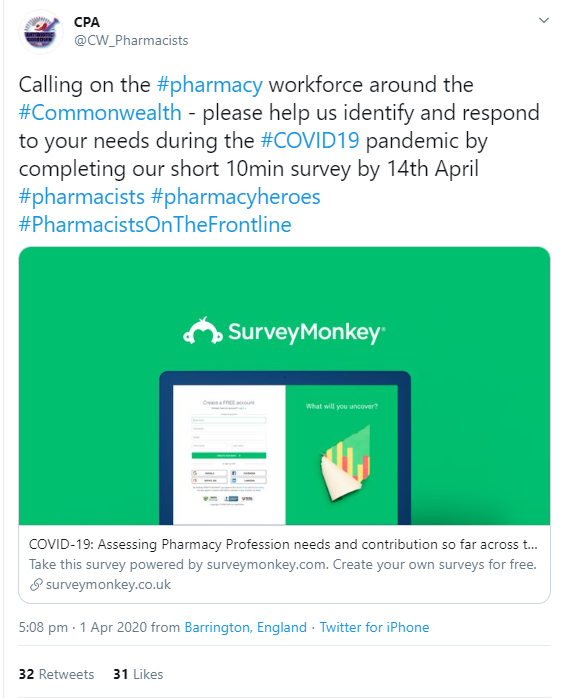  <https://twitter.com/CW_Pharmacists/status/1245382644994637824> | 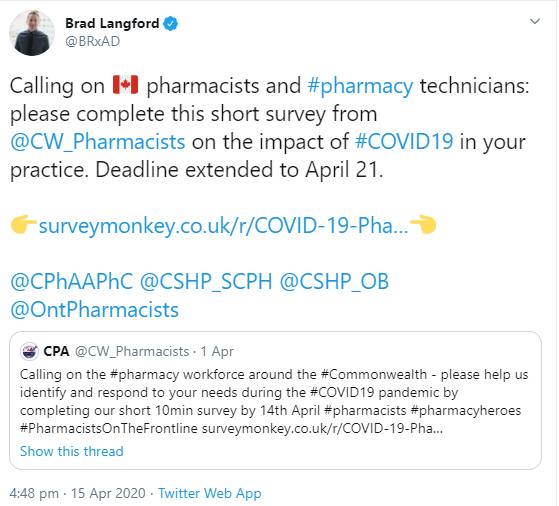#  <https://twitter.com/BRxAD/status/1250451058226331649?s=20> |
| --- | --- |
