# Supplementary material for: Needs assessment and impact of COVID-19 on pharmacy professionals in 31 commonwealth countries
Source: J Pharm Policy Pract. 2020 Oct 21;13:72. doi: 10.1186/s40545-020-00275-7 (PMC7576098; doi:10.1186/s40545-020-00275-7)
Supplement: Supplementary file 2 — Additional file 2. CPA impact of COVID-19 and Pharmacy Needs Assessment Survey Tool. [file 40545_2020_275_MOESM2_ESM.pdf]

COVID-19 response

We invite all pharmacy professionals and pharmacy professional bodies in the commonwealth to complete this survey which is opened initially until 14 April 2020 (NOW EXTENDED to 21 April 2020).
The purpose of this survey is to understand:
*** what issues are affecting pharmacy professionals' ability to respond to the COVID-19 pandemic,**
*** how pharmacy professionals and professional bodies are responding to the pandemic and**
*** what support you believe would be helpful from the Commonwealth Pharmacists Association or other pharmacy bodies**

The survey should take approximately 10 minutes to complete.

All pharmacy professionals (pharmacists, pharmacy technicians, dispensers) as well as pharmacy professional bodies are encouraged to complete the survey. Kindly share the survey link with other pharmacy colleagues

Please try to answer all questions so we can understand need and how to support or advocate for support. We will aim to publish the findings to share knowledge and support advocating for the profession.

Thank you in advance for your important contribution
Dr Diane Ashiru-Oredope
On behalf of the Commonwealth Pharmacists Association Team

* 1. The survey is completely voluntary and part of CPA's service provision. You have the right to refuse to answer questions or withdraw at any time.
By proceeding to the next page:

- I consent to CPA collecting and using the information about me that I voluntarily provide for the purposes of the survey
- I have read, understand and agree to the information provided above

◯ YES, I am happy to proceed with the survey

◯ NO, I am not happy to proceed with the survey and I do not wish to continue

* 2. In what country do you work?

* 3. What is your profession?

- Pharmacist
- Pharmacy technician
- Pharmacy student
- Other (please specify)

4. Gender

- Female
- Male
- Other
- Prefer not to say

* 5. In which sector do you predominantly work in? E.g. If you work in a community pharmacy and hospital - choose the one you spend most of your time in

- Community
- Hospital
- Public health institute
- Academia
- Professional body
- Government - local, regional or national
- Other (please specify)

6. Are you responding as an individual, on behalf of a pharmacy professional body/organisation or both? (please select one only)

- ◯ Myself
- ◯ Pharmacy professional body/organisation only
- ◯ Both
- ◯ Other (please specify)

7. Which pharmacy professional group(s)/organisations do you belong to? E.g. Pharmaceutical Society of xxxxx

8. If you are including responses on behalf of a pharmacy professional body/organisation - please state full name (including country)

Name of professional body

Country

Your role in the professional body (e.g. secretary, president, professional body's office team)

9. How worried are you about the impact of COVID-19 on you personally?

- ◯ Extremely worried
- ◯ Very worried
- ◯ Somewhat worried
- ◯ Not so worried
- ◯ Not at all worried

* 10. How worried are you about the impact of COVID-19 on pharmacy profession in your country?

- ◯ Extremely worried
- ◯ Very worried
- ◯ Somewhat worried
- ◯ Not so worried
- ◯ Not at all worried
- ◯ Other (please specify)

11. How easy or difficult is it for you to work effectively these days?

- Very easy
- Somewhat easy
- Neither easy nor difficult
- Somewhat difficult
- Very difficult

12. Have you had to work more remotely as a result of COVID-19?

- Yes
- No
- Partly

Other (please specify)

13. If you are having to work more remotely, what are the TOP THREE biggest challenges you are currently facing while working?

- [ ] My physical workspace
- [ ] Too many distractions at home
- [ ] I don’t have access to the tools or information I need to do my job at home
- [ ] Internet connectivity
- [ ] Childcare
- [ ] Social isolation
- [ ] Communication with coworkers is harder
- [ ] I’m sick or helping others who are sick
- [ ] Keeping a regular schedule
- [ ] General anxiety about the impact of coronavirus on my life
- [ ] Getting enough food
- [ ] Other (please specify)

14. What is your single greatest work-related concern right now/for the future as a result of COVID-19 impact?

15. Has your pharmacy organisation or a pharmacist you know been involved/consulted in COVID-19 response or preparation? (please select only one option per row)

	Directly	Peripherally (indirectly)	No involvement	Other	Don't know
Pharmacy Organisation	◯	◯	◯	◯	◯
Pharmacist (yourself)	◯	◯	◯	◯	◯
Pharmacist (another colleague)	◯	◯	◯	◯	◯

Please provide further details

16. Has there been any COVID-19 response in your country which was spearheaded/proposed by the pharmacy organisation in your country?

◯ Yes

◯ No

◯ Unsure

◯ Sort of

Please provide further details

17. Please share your views on COVID-19 implication for pharmacy in your country and/or how you believe the pharmacy profession can be of added value/support

18. Global/public health emergency preparedness

	Yes	No	Partly	Unsure	N/A
Have you previously received any training on emergency preparedness	◯	◯	◯	◯	◯
Please provide further details, eg. type of training					
Have you previously been actively involved in a global health emergency preparedness eg tackling Ebola?	◯	◯	◯	◯	◯
Please provide further details, eg. type of training					

19. What support would you find helpful from CPA at this time? (select all that apply)

- [ ] Webinars on Covid 19
- [ ] Access to a community of support to share questions and concerns
- [ ] Signposting to information
- [ ] others (please specify)

20. What other concerns do you have - nothing is too insignificant to share

21. What sources of information have you used so far to learn about COVID-19? (select all that apply)

- [ ] WHO
- [ ] Africa CDC
- [ ] National Ministry of health / Local health authority
- [ ] Professional associations
- [ ] US CDC / European CDC
- [ ] University / Hospital
- [ ] Internet search (Google, YouTube)
- [ ] Radio
- [ ] Television
- [ ] Professional network
- [ ] I did not look for guidance
- [ ] Other (please specify)

22. Have you undertaken any training courses or joined any training webinars from providers?

- [ ] WHO
- [ ] Africa CDC
- [ ] Ministry of health / Local health authority
- [ ] Professional associations
- [ ] CDC / ECDC
- [ ] University / Hospital
- [ ] I have not taken any courses
- [ ] Other (please specify)

23. Which of the following areas on COVID-19 would you want to increase your knowledge of?

- [ ] Clinical management of cases
- [ ] Pharmaceutical management of COVID-19
- [ ] Infection prevention control
- [ ] Use of personal protective equipment
- [ ] Epidemiology
- [ ] Risk communication
- [ ] Operational support and Logistics
- [ ] Research & development
- [ ] Water, hygiene and sanitation
- [ ] I don't need to improve my skills
- [ ] I am unsure
- [ ] Other (please specify)

Social distancing and impact on pharmacy workload

24. Have social distancing measures (e.g. school closures, gatherings cancelled etc) been introduced in your country? (please select one that best describes current situation)

- ◯ Yes - broadly mandated
- ◯ Sort of - encouraged
- ◯ No
- ◯ Not sure

25. If social distancing has been introduced, how is this affecting the pharmacy profession in your country?

- ◯ Significantly increased workload and visits to the pharmacy
- ◯ A slight increase in workload and visits to the pharmacy
- ◯ Reduced workload and visits to the pharmacy
- ◯ Not much impact on workload or visits to the pharmacy
- ◯ Don't know
- ◯ Other (please specify)

26. Please share examples of what measures have been introduced in pharmacy settings as a result of COVID-19 e.g. limiting number of customers in the pharmacy, screens, use of PPE?

27. Please share examples of international support which have benefited health teams in your country. Eg provision of PPE, training, support for hand hygiene etc

About you

* 28. Years of experience in current profession

- Less than 1 year
- 1- 3 years
- 4 - 10 years
- 11 - 15 years
- > 15 years

29. Age?

- 18 to 24
- 25 to 34
- 35 to 44
- 45 to 54
- 55 to 64
- 65 to 74
- 75 or older
- Prefer not to say

30. Please provide website URL for the professional body/organisation (if you included responses on their behalf)

31. If you would like to be informed of any relevant information on COVID-19 (e.g. summary of this survey, notification of webinars), provide your email address below

32. Please use this section to share any relevant comments
